# Practical Considerations in the Radiotherapy Treatment Planning for SBRT Pancreas Program

**DOI:** 10.3390/curroncol33090519

**Published:** 2026-08-31

**Authors:** Kurian Joseph, Ben Burke, Amr Heikal, Eugene Yip, Shannah Murland, Clarence Wong, Beena Kunheri

**Affiliations:** 1 Department of Oncology, University of Alberta, Edmonton, AB T6G 1Z2, Canada; ben.burke@cancercarealberta.ca (B.B.); amr.heikal@cancercarealberta.ca (A.H.); eugene.yip@cancercarealberta.ca (E.Y.); 2 Division of Radiation Oncology, Cross Cancer Institute, Edmonton, AB T6G 1Z2, Canada; shannah.murland@cancercarealberta.ca; 3 Division of Medical Physics, Cross Cancer Institute, Edmonton, AB T6G 1Z2, Canada; 4 Division of Gastroenterology, Department of Medicine, University of Alberta, Edmonton, AB T6G 1Z2, Canada; clarence.wong@albertahealthservices.ca; 5 Kent Oncology Center, Maidstone and Tunbridge Wells NHS Trust, Maidstone, ME16 9QU, UK; beena.kunheri@nhs.net

**Keywords:** pancreatic carcinoma, radiotherapy, stereotactic body radiotherapy

## Abstract

Pancreatic ductal adenocarcinoma is one of the most aggressive tumours, with an estimated 5-year overall survival rate of 5% and median survival of 5–11 months. Approximately one third of patients die of complications due to local disease progression, especially among patients with borderline resectable or locally advanced disease; hence achieving local control is significant for improved survival outcomes. Stereotactic body radiotherapy (SBRT) allows safe and effective delivery of ablative doses of radiation and is associated with improved locoregional tumour control and progression free survival. Our article describes the rationale and the safe and effective delivery of Linac-based SBRT treatment for pancreatic cancer.

## Simple Summary

Pancreatic cancer (PC) is the eleventh most common cancer in the world and is the seventh leading cause of global cancer deaths. The exact cause of PC is not well defined. About 5–10% of patients are associated with hereditary pancreatic cancer syndrome. The most common histological type is pancreatic ductal adenocarcinoma (PDAC), and about 70% of PC arise in the head of pancreas. The stereotactic body radiotherapy (SBRT) program for pancreatic cancer is a relatively novel treatment approach typically offered for patients with non-metastatic pancreatic cancer who are unresectable even after completion of systemic therapy. Multiple single institutional studies have reported that SBRT is a promising treatment option to achieve local control with limited acute toxicities. Currently, the phase III NRG-GI011 trial is evaluating whether SBRT improves survival for locally advanced, unresectable pancreatic cancer.

## 1. Introduction

Pancreatic cancer (PC) is the eleventh most common cancer in the world and is the seventh leading cause of global cancer deaths [1]. The exact causes of PC are not well defined and some of the risk factors identified include family history, smoking, chronic pancreatitis, diabetes mellitus, alcohol, and obesity [2]. About 5–10% of patients are associated with hereditary pancreatic cancer syndrome [3]. Approximately 90% of PC are exocrine and the most common histological type is pancreatic ductal adenocarcinoma (PDAC). Almost 70% of PC arise in the head of pancreas, 15% in the body, and 15% in the tail. Early diagnosis of pancreatic cancer remains challenging.

The definite management of PDAC is challenging and the outcomes of treatment largely depend on the stage of the disease. In general, patients are divided into four categories for the purpose of selecting specific treatment based on the degree of tumour contact and invasion into the superior mesenteric, hepatic, or celiac vessels. The different categories are resectable PDAC, borderline resectable PDAC (BRPC), locally advanced PDAC (LAPC), and metastatic. Resectable PDAC are tumours without vascular invasion whereas borderline resectable PDAC (BRPC) are tumours with involvement of vascular or local structures or high risk of R1 resection, and are associated with high morbidity and high early systemic recurrence [4,5]. Locally advanced PDAC (LAPC) are not operable due to vessel invasion but are non-metastatic tumours.

Surgical resection is the only definitive treatment that offers a potential cure. Whipple’s procedure (pancreatico-duodenectomy) or distal or total pancreatectomy are the surgical procedures typically used, depending upon anatomical location of the tumour. About 15–20% of highly selected patients are resectable, whereas 30–40% are BRPC or LAPC at initial presentation. Although early-stage disease can be treated with surgery, the prognosis is poor since the risk of local and distant failures can be as high as 37% and 75%, respectively. Most failures occur within one-year post-surgery with a median overall survival of approximately 20 months [6]. The landmark CONKO (Charité Onkologie Clinical Studies in GI Cancer)-001 trial reported improved disease-free survival in patients who received adjuvant gemcitabine following complete tumour resection compared to those who received surgery alone (13.4 months vs. 6.7 months) [7]. The randomized phase III study by the PRODIGE24/CCTG group reported that adjuvant therapy with modified FOLFIRINOX regimen was associated with significantly improved disease-free survival (21.6 months vs. 12.8 months) and overall survival (54.4 months vs. 35 months) compared to gemcitabine among patients with resected pancreatic cancer [8]. Hence, surgical resection followed by adjuvant chemotherapy with modified FOLFIRINOX regimen is the standard of care for resectable PC.

At present, there is no consensus on the role of neoadjuvant or adjuvant radiotherapy for the treatment for BRPC or LAPC, which accounts for one-third of newly diagnosed PC. About 30% of patients with BRPC or LAPC could die of complications due to local disease progression; hence local disease control may result in significant improvement in survival [9]. Therefore, treatment of BRPC or LAPC may involve a combination with radiotherapy (RT) and could lead to improvement of local control. However, the role of RT remains controversial due to conflicting data from various studies. The results from various studies using adjuvant or neo-adjuvant RT are given in Table 1.

Various phase I/II single institutional studies reported that the addition of neo-adjuvant radiotherapy to systemic therapy in the treatment of BRPC or LAPC has converted about 10–30% of patients to resectable disease without survival improvement [12,16,17,18,19]. BRPC or LAPC has a high risk of R1 resection and possible occult metastasis. The neo-adjuvant approach for the treatment of BRPC or LAPC has reported many advantages. The approach would sterilise undetected micrometastases and increase R0 resection rate; however, delayed resection could reduce the chance of cure in BRPC. Conventional CRT was associated with substantial treatment-related toxicity. Hence, in general, patients with BRPC or LAPC are treated with neo-adjuvant FOLFIRNOX or gemcitabine/nabP chemotherapy initially (usually 2–6 months) followed by surgery.

Stereotactic body radiotherapy (SBRT) involves a short course (<5 fractions) of radiation with an ablative dose (>6 Gy per fraction) and has demonstrated a high rate of tumour necrosis through its ability to prevent endothelial and micro-vascular stabilization [20].

No prospective data comparing SBRT to conventional fractionated radiotherapy for the treatment of pancreatic cancer is available at present. Reyngold et al. from the Memorial Sloan Kettering Cancer Center have reported that patients with LAPC treated with SBRT following multiagent induction chemotherapy were associated with improved locoregional tumour control and progression free survival [21]. Retrospective data from multiple single institutional studies reported the safety and efficacy of multifractional SBRT. A systematic review and pooled analysis of 19 trials with 1009 LAPC patients indicated that SBRT is safe with <10% late gastrointestinal toxicity and local control of 73% at 1 year [22]. To promote low incidence of toxicities, the commonly adopted SBRT dose regimen is 33 Gy delivered in five fractions over 1–2 weeks, which is equal to 54.78 Gy BED using the standard linear-quadratic conversion. Hoyer et al. reported treating 22 patients with SBRT using a dose of 45 Gy in three fractions (BED 112.5 Gy for alpha/beta = 10) [23]. The study reported a local control of 57% at 6 months, but with significant acute and late toxicity (79% and 94% respectively). Retrospective evidence suggest that a biologically effective dose (BED) of at least 70 Gy may be beneficial in the treatment of PDAC [24]. Reyngold et al. from MSKCC has recommended using SBRT for dose-escalation with BED ≥100 Gy to achieve high tumour control with minimal acute treatment related toxicity [25]. This planning strategy would also enable as much of the tumour as possible to receive the prescribed dose while restricting the dose in the areas directly abutting the organs at risk (OARs) to safe limits. There is an increase in interest in the use of SBRT for pancreatic cancer for those patients who are not operable after systemic therapy and without evidence of disease progression. The indications for considering SBRT treatment instead of conventional 3-D conformal chemoradiation are small tumours (≤6 cm), preferably located ≥1 cm away from gastrointestinal mucosal organs and without evidence of positive lymph node involvement [26].

For the effective and safe delivery of ablative doses through techniques such as SBRT, a number of factors should be considered, including anatomic considerations, dose heterogeneity, and technology use (e.g., image guidance). SBRT uses robust immobilization, motion management, and conformal dosimetry to deliver a high dose per fraction to increase the BED of radiation treatment that leads to improved local control with reduced incidence of acute and late toxicities [26]. SBRT can be performed either by CyberKnife Robotic Radiosurgery (Accuracy Inc., Sunnyvale, CA, USA), by linear accelerator (Linac)-based platforms such as Trilogy and TrueBeam (Varian Medical Systems, Palo Alto, CA, USA), or by stereotactic magnetic resonance-guided adaptive radiotherapy (SMART) using Linac-MRI. The basic principles are the same for these techniques. This paper primarily focusses on treatment planning and delivery for linac-based SBRT based on our experience as well as consultation with experts on SBRT.

## 2. Patient Selection

The primary objective of SBRT in pancreatic cancer is to deliver dose-escalated RT for possible improvement in local control and survival in patients who are treated initially with chemotherapy but are inoperable without disease progression. This approach could result in possible down-sizing and downstaging of the non-operable tumour and allow surgical removal [27]. However, SBRT can also be offered for symptom palliation. Hence, patient selection for SBRT is clinically important and vital for the success of the treatment. In general, patients considered for SBRT should have good performance status (ECOG 0–2 or Karnofsky performance status ≥ 70) and life expectancy > 6 months. Most of the multi-institutional SBRT studies included only patients who have received 4–6 months of induction or neo-adjuvant chemotherapy with FOLFIRINOX or NALIRIFOX or gemcitabine/nab-paclitaxel and are inoperable without disease progression (Supplementary Table S1) [28,29,30,31,32,33,34,35,36,37,38,39,40,41]. If the baseline CA19.9 is elevated, then the pre-SBRT CA19.9 value must be less than 50% of the pre-chemotherapy level [42]. The required tumour characteristics include tumour size limited to <6 cm and well controlled or absent extra pancreatic disease. Other requirements include adequate hematologic function (absolute neutrophil count (ANC) ≥ 1500 cells/mm^3^, platelets ≥ 70,000 cells/mm^3^, hemoglobin ≥ 8.0 g/dL and normal INR/PT), liver function (AST & ALT < 2.5 × upper limit, total bilirubin < 1.5 ULN). Treatment response can be monitored by CA 19.9 levels. Tumours with vascular invasion are not a contraindication for SBRT. SBRT is usually contraindicated when there is tumour invasion into the duodenum or other GI structures and patients are generally excluded if they have nodal involvement or metastatic disease.

Patients eligible for SBRT should be discussed by a SBRT multidisciplinary tumour board and group consensus achieved before proceeding with treatment.

## 3. Fiducial Insertion

Fiducial placement provides safe and accurate targeting of the tumour during SBRT [43]. Ideally, the first step in the SBRT process is endoscopic insertion of MRI compatible fiducial markers into the target lesion in the pancreas. Fiducials are radiographically visible markers that allow better tumour localization and tumour tracking during treatment delivery [44]. At least three fiducial markers should be placed in different endoscopic ultrasound (EUS) viewing planes so that the pancreatic tumour borders and planes can be well defined during SBRT simulation and delivery [45]. Fiducials should be placed at the periphery of the tumour, when possible, to have ample distance between fiducials. Biliary stent insertion is not mandatory unless the patient has signs of obstruction.

## 4. Respiratory Motion Assessment

The goal of the motion assessment is to reduce the motion of the tumour to less than 5 mm. The pancreas is susceptible to motion caused by breathing and motion mitigation is a vital component of pancreatic SBRT to allow for target volumes to be reduced and help minimise dose to adjacent OARs [46].

**Figure 1:**
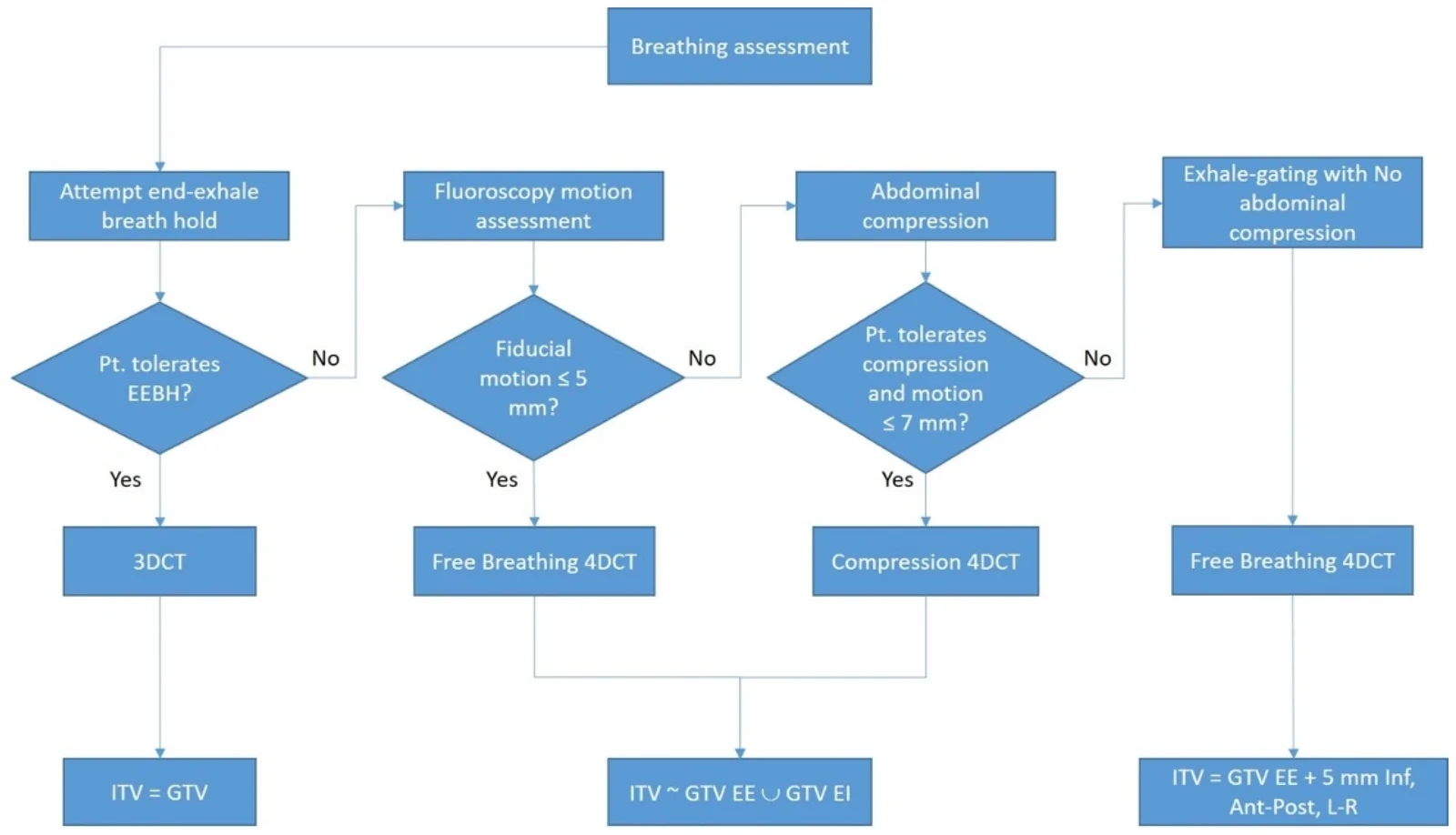
Flowchart showing the impact of respiratory motion in the target definition for SBRT pancreas. EEBH: end expiratory breath hold, 3DCT: 3-dimensional CT scan, 4DCT: 4-dimensional CT scan, GTV: gross tumour volume, ITV: internal target volume.

Figure 1 shows an overview of the decision-making process for the motion assessment. If the fiducial motion with the patient in breathing normally is <5 mm, the patient can be treated with the free-breathing (FB) protocol, however, this is a rare occurrence and most patients require further motion management. A respiratory motion assessment is to be completed before CT simulation, a minimum of five days after fiducial insertion. Our institution schedules a motion assessment appointment on the treatment unit just prior to CT simulation.

**Figure 2:**
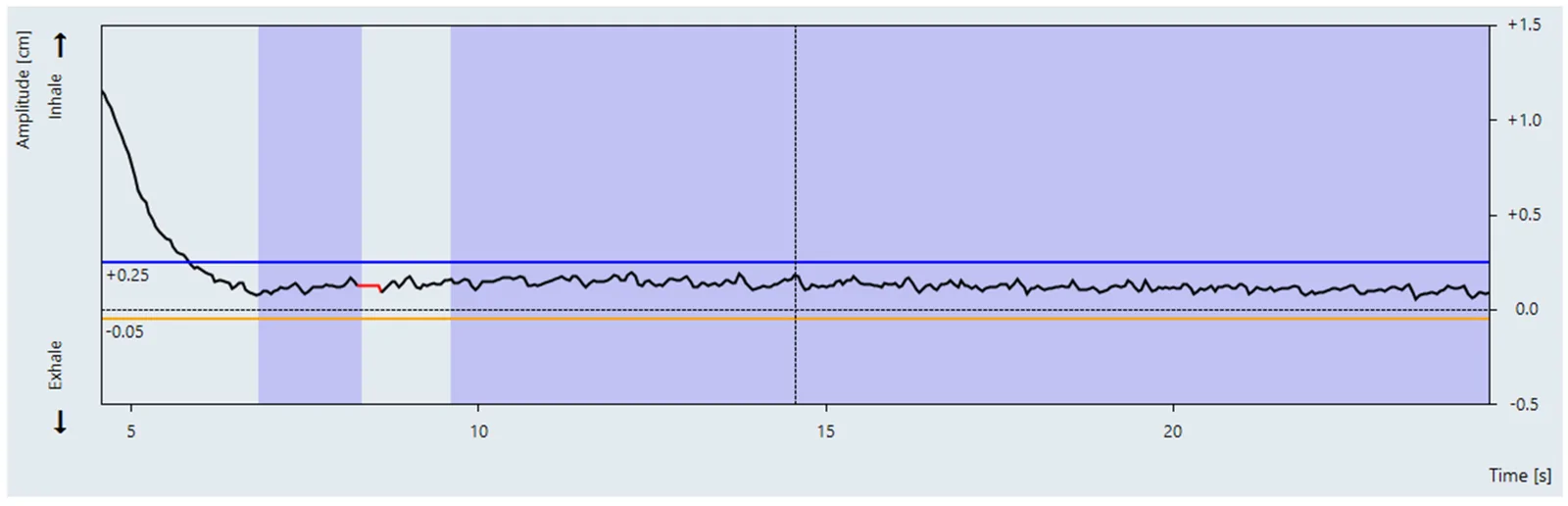
Real time respiratory trace illustrating an acceptable breath-hold. Blue and orange line defines a 3 mm breathhold window. If the breathing trace drifts out of this window, the beam is stopped.

Our institutional practice is to have the patient lay on the treatment unit couch, and the immobilization system, comprised of a Vac-Fix bag on an indexed board, is formed on the spot with the patient’s arms above their head for gantry and beam clearance. Once complete, the on-board kV imaging device is used to acquire fluoroscopy images where the fiducials are visualized, and their total motion can be measured with the patient free-breathing and/or with abdominal compression applied. The patient’s normal breathing pattern is captured by placing a respiratory block (Figure 2) on the patient’s abdomen and allowing the in-room IR camera system to capture a respiratory trace. The patient is instructed to hold their breath, and the length and stability of the breath-hold is evaluated. Fluoroscopy and the breathing trace are used to visualize fiducial motion and to choose the best means of breathing motion management, with the goal of minimizing target motion.

There are multiple methods that can be used for motion management. End Expiration Breath Hold (EEBH) is the preferred motion management strategy as it provides maximum motion reduction. The patient is instructed to hold their breath in their end-expiration phase and the respiratory trace is evaluated. Acceptable EEBH requires that the patient be able to hold their breath, reproducibly, for a period equivalent to at least the length of the simulation scan, generally 15–25 s [47]. However, the longer the breath-hold that can be achieved, the more efficiently the treatment can be delivered, and the patient will not have to be in the treatment position for as long. They must be able to follow instructions from the radiation therapists, who play a key role in assessing patient suitability and helping the patient achieve the same exhale position reproducibly. EEBH provides improved stability for the target and allows more conformal OAR-sparing treatment delivery. If EEBH is not tolerated or deemed non-reproducible, abdominal compression or gating will be evaluated using the fluoroscopy imaging.

Abdominal compression can be used to reduce target and OAR motion significantly in most patients. Our institutional guidelines are to try and reduce the motion to less than 5 mm; however, a residual motion of up to 8 mm may be accepted. Breathing while under abdominal compression also requires patient education such that the patient is taught how to breathe with their chest and does not resist the compression. A potential disadvantage of abdominal compression is that compression may displace OARs towards the primary target volume [48]. In addition, the day-to day reproducibility and effectiveness of compression could vary between fractions, and hence daily image guidance is required.

If neither of the above methods are possible, the patient may be treated with respiratory gating based on breathing motion. The patient’s free-breathing trace must be evaluated to ensure that their breathing has consistent amplitude and rate. The patient will be treated in the phases surrounding end-exhalation, which limit the motion to a specified value; at our institution this is 5 mm. Gating can be difficult due to challenges in reproducing the breathing rhythm for each treatment and an inefficient duty cycle. Because the patient is treated in short intervals only at exhale, treatment duration can be quite long.

## 5. Patient Set up for CT Simulation

CT simulation for pancreatic SBRT involves creation of 3D or 4D images to precisely define the tumour and surrounding OARs. Patients are required to fast for 4 h before the breathing assessment, CT simulation and treatment. Requiring the patient to have an empty stomach helps to stabilize the position of the target and nearby GI structures and minimize daily shape changes. The patient is immobilized in a supine position in a Vac-Fix bag or similar custom-made device with arms above the head to have better clearance of gantry angle, and a knee rest in a comfortable position. If the patient cannot achieve a position with arms overhead, the position can be adapted for one or both arms by sides.

## 6. CT Simulation

CT simulation typically occurs on the same day after the breathing motion assessment, and the scan length should extend from 5 cm above the diaphragm to the bottom of L5. The patient is requested to be NPO (nothing by mouth) with instructions not to eat or drink for 4 h prior to simulation (as well as for treatments). However, the patient is allowed to take oral contrast during simulation, and medications with clear fluid to help with pain, nausea, or vomiting before treatment if required. All CT scans are acquired using a 2 mm slice thickness. A 3DCT scan is acquired if EEBH is selected as the motion management strategy and a 4DCT is acquired for all other motion management techniques. If contrast is required, the timing and volume of contrast depend on the contrast agent and the structures to be visualized. We use 60–65 mL of dilute oral contrast (15 mL Omnipaque300 diluted in 50 mL water) that is given to the patient 10–15 min prior to scanning, and use Omnipaque300 as IV contrast that is dosed according to weight; typically 98 cc is administered at 3 mL/sec. This improves tumour visibility and visualization of the duodenum and as well as displacing the lateral wall of the duodenum.

Patients should be coached for timing their breath-hold with the contrast delay and start of the scan. For EEBH, the 3DCT is performed with the appropriate scan delay according to the CT sim procedures (~45 s delay) from the start of injection for pancreatic phase.

A specialized in-house contrast delay calculator is used for 4DCT scans and is based on the work of Helou et al. [49]. A 4DCT is performed for free breathing, abdominal compression, or gated treatments.

## 7. MR Simulation

MRI simulation is done in the treatment position once CT simulation is completed. MRI sequences include T2-weighted, half Fourier single shot Turbe Spin Echo for bile duct visualization, and post-contrast T1-weighted, fat suppressed breath-hold images for tumour visualization (HASTE, VIBE-FS). Contrast-enhanced MRI sequences, which best visualize the tumour, are obtained at end expiration and are registered with the end expiration 3DCT scan in the EEBH scenario. For abdominal compression and FB (free-breathing) treatments, an additional end inspiration MRI visualizing the tumour is acquired, and the expiration/inspiration MR images are registered with end expiration/ inspiration phase of the 4DCT, respectively, to help generate an Internal Target Volume (ITV). The oncologist will review the diagnostic MRI to determine the need for contrast with the planning MRI. If the tumour is well visualized without gadolinium, the MRI will be performed without contrast. If the patient is not having an MRI, they should receive IV contrast at the time of CT simulation.

## 8. Target Contouring

The current recommendations on target delineation are based on a study by Herman et al. and the NRG-G1011 protocol [NCT06958328] [28,50]. The gross tumour volume (GTV) includes all gross tumour seen on CT or MRI. If a 4DCT has been acquired, GTV contouring will be performed on the average scan and not on the maximum intensity projection (MIP). The average scan should include all phases where the beam is turned on (i.e., all phases for FB or compression and phases within the gating window for gated treatments). Registration of the end inspiration and end expiration sequences of the planning MRI with the corresponding phases is essential if 4DCT is being used. GTV-Inspiration and GTV-Expiration will be contoured on each of the appropriate phases of the respiratory motion. For EEBH, the GTV is contoured on the registered end-exhale MRI and 3DCT. Typically, elective nodal regions are not included in the target. An upper abdominal/liver atlas, posted on the RTOG website, may be used as a guide for contouring [50].

**Figure 3:**
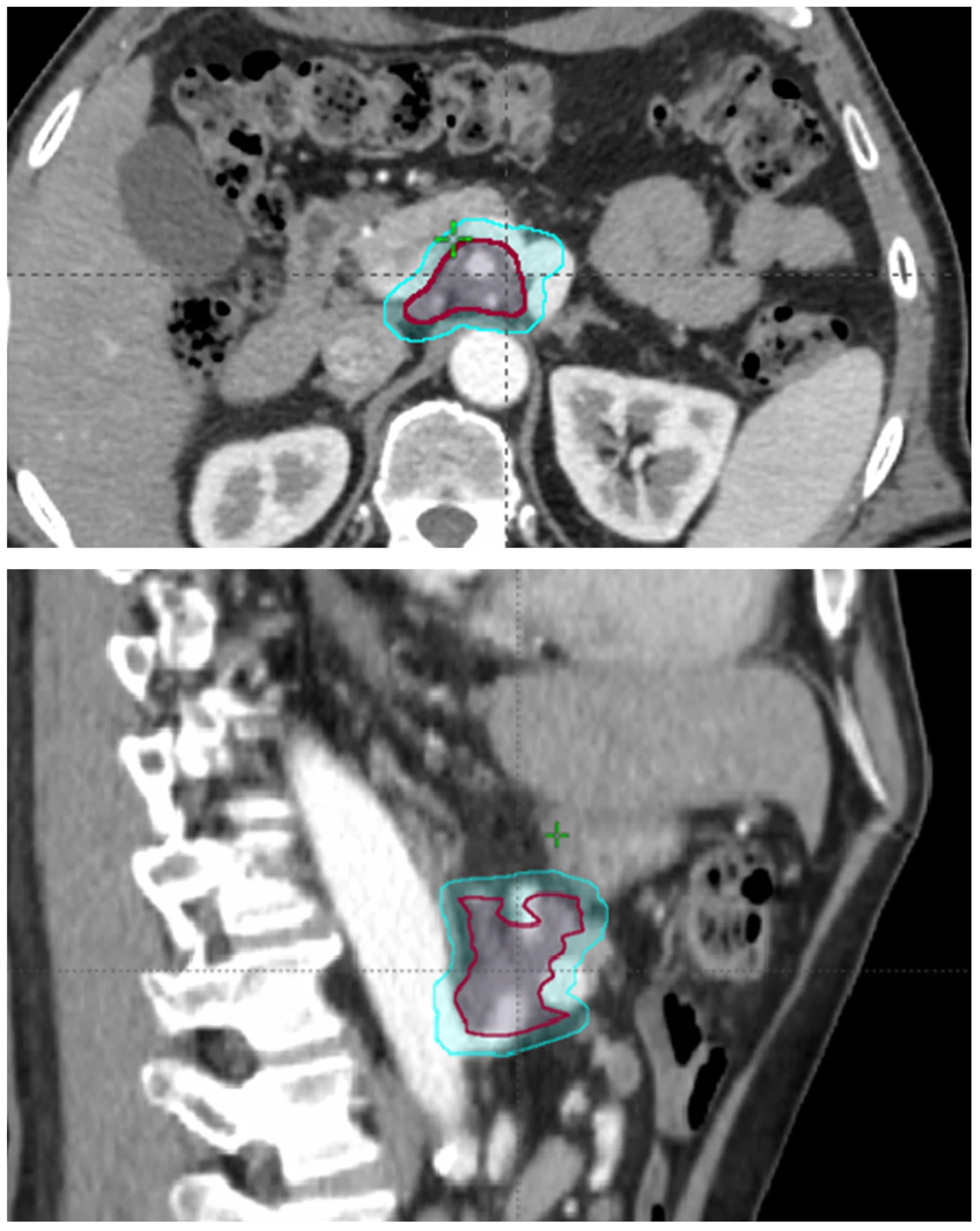
Axial and sagittal view of a patient simulated in EEBH. No ITV required. PTV (cyan) is created with a margin of 3 mm around GTV (red).

**Figure 4:**
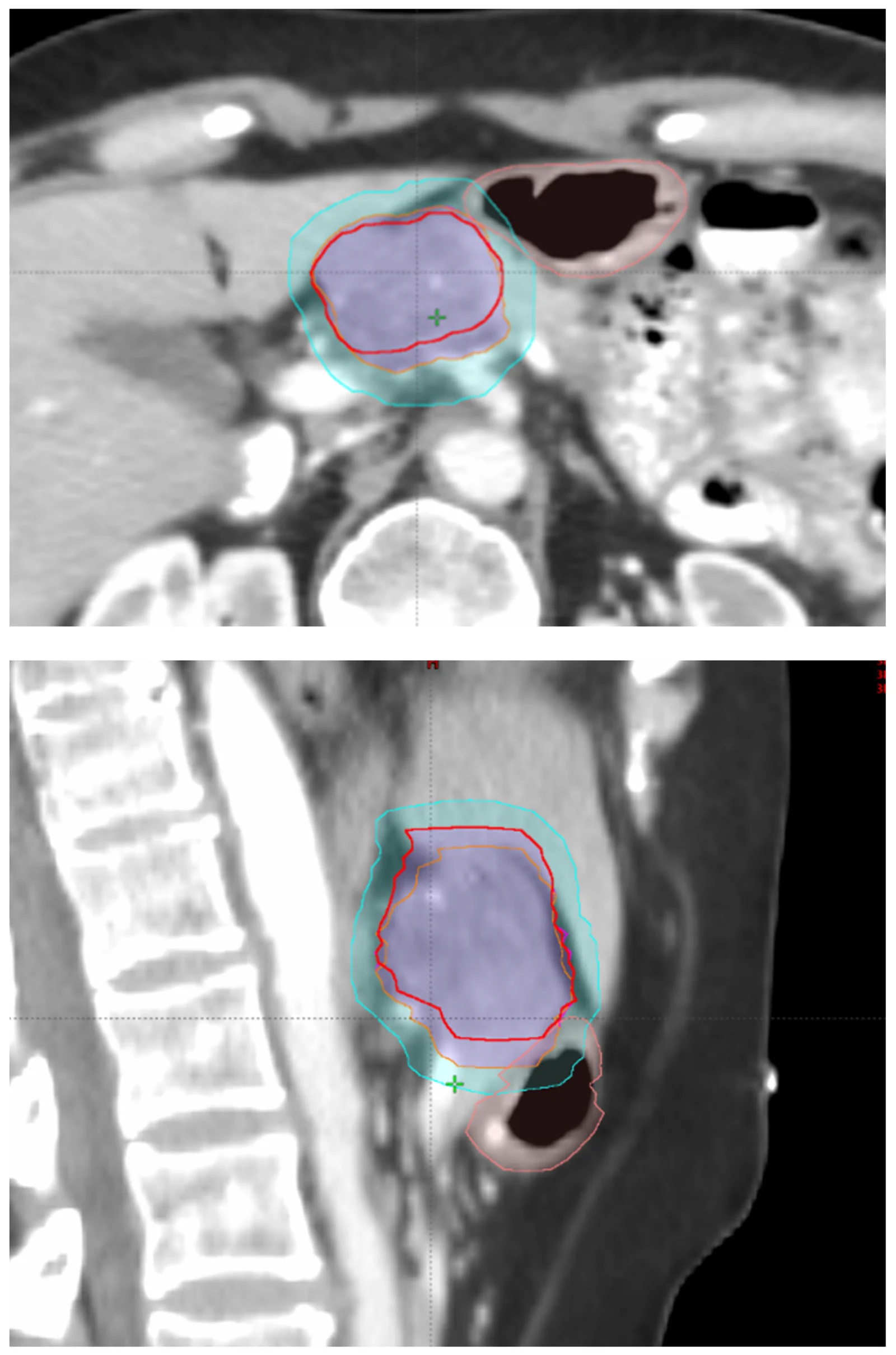
Axial and sagittal views of a patient simulated with abdominal compression. GTV-Expiration (red) and GTV-Inspiration (orange) combined to create ITV (shaded area in violet). PTV (cyan) is created with a margin of 3 mm around ITV (violet). Duodenum (pink).

The Internal Target Volume (ITV) is the volume encompassing the GTV and accounts for GTV movement caused by breathing, which can also lead to internal organ motion with respect to position, shape, and size changes [51]. For FB and abdominal compression treatments, the ITV is the union of the GTV inspiration and GTV expiration that incorporates all phases of respiratory motion. For gated treatments, the ITV accounts for motion within the gating window. At our institution, the gating window surrounds end exhalation, so there is no longer a GTV-inspiration and a different ITV strategy is required. One straightforward strategy for defining the ITV is to symmetrically expand the GTV-expiration by 3–5 mm in all directions except superiorly to generate an ITV. The ITV is not grown in the superior direction as the end expiration phase will be the most superior position of the liver and target, so will not require further expansion. The magnitude of expansion depends on the selection of the gating window. Our institution selects the phases surrounding end exhalation, which limit motion in any direction to less than 5 mm, thus applying a 5 mm expansion. Alternatively, one can define a custom ITV expansion based on the motion of the fiducials within the gating window as measured on the 4DCT. Finally, one can also contour the GTV in all phases within the gating window and combine to create the ITV. For EEBH, the ITV is the same as the GTV because there is no motion to account for. The Planning Target Volume (PTV) is a geometrical margin that allows for uncertainties in treatment planning and delivery [52]. The PTV is generated by adding a margin of 2–3 mm around the ITV or the GTV for patients treated with EEBH [28,53]. The selection of margins is dependent on the availabilities of motion management technologies (Figure 3 and Figure 4). The ITV generation methods based on their respective motion management technique of our institution is summarized in Table 2.

Organs at risk (OARs) are normal structures whose radiation sensitivity influences treatment planning and the prescription dose. Hence, there will be dose constraints for each OAR. Both systematic and random errors apply to OARs just as much as to the CTV, and so a minimum margin of 3 mm expansion around the stomach, duodenum, small bowel, and large bowel is used to generate a planning organ at risk volume (PRV), analogous to the PTV margin around the CTV [54].

The fiducials are contoured to include all phases of the respiratory cycle; this can be achieved by using a maximum intensity projection (MIP) or by contouring the phases individually and combining into a single structure. For EEBH patients, the fiducials are contoured as seen on the 3D scan. A margin (e.g., 3 mm) can be added to the fiducial contour to define a “motion contour” that can be used for real time verification of tumour position during treatment using kV imaging (Auto Beam Hold, TrueBeam 2.0 & 2.5, Varian Medical Systems).

Our SBRT program strongly recommends peer review of all the contours by a second radiation oncologist before sending for planning as well as review of the plan and normal tissue constraints prior to therapy.

## 9. Radiation Dose and Treatment Schedule

SBRT involves a short course (<5 fractions) of radiation with an ablative dose (>6 Gy per fraction). The most common SBRT dose and fractionation schedule used is 33 Gy in five fractions (33–40 Gy delivered in five fractions of 6.6 Gy to 8 Gy) [28]. Our dose regimen varies from 35–50 Gy in five fractions based on multiple patient and treatment planning related factors, and at the discretion of the treating physician. The significant predictors that could be affecting dose/prescription include distance of PTV from critical structures such as stomach or duodenum, type of respiratory motion management, planning technique and institutional tolerance risk data. Closeness to target could be the prime factor compromising the dose/prescription. However, many institutions with MRI-guidance or adaptive radiotherapy facility routinely prescribe 50 Gy in five fractions that would provide dose-escalated RT with BED ≥ 100 Gy, and aim to achieve higher tumour control [55].

In general, the time between fractions should be between 24 and 72 h, with treatment delivered to all targets over 2 weeks. The preferred interfraction interval is 48 h. For a five-fraction schedule, a typical schedule would be Monday, Wednesday, and Friday, completed over 2 weeks.

## 10. Critical Normal Structures

Dose-escalated SBRT (DE-SBRT) has reported improved survival compared to standard doses [24]. However, SBRT doses for pancreatic cancer have been limited to subtherapeutic levels due to closeness of OARs to the target. Tumour motion is also a major source of uncertainty in SBRT to the pancreas. The primary OARs are small bowel, duodenum, stomach and large bowel and avoidance of GI toxicity is a prime consideration in SBRT planning. Generally, OARs are contoured 2 cm above and below the PTV such that they can be properly evaluated on the DVH. A minimum expansion of 3 mm around the stomach, duodenum, small bowel, and large bowel can be used to generate a planning organ at risk volume (PRV) to account for daily setup uncertainty and internal organ motion that will help to tighten high-dose gradients and safely spare adjacent OARs. In general, most RT departments maintain an institutional OAR dose constraints protocol for safe SBRT planning and treatment delivery. The Australasian trials group, along with the Trans-Tasman Radiation Oncology group and ASTRO, have defined constraints for organs at risk for planning pancreatic SBRT for Linac-based platforms [52,53]. Grimbergen et al. reported a global consensus protocol for treatment planning for MR-guided SBRT [55]. Table 3 describes dose constraints recommended for SBRT planning, depending on different fractionation schedules.

## 11. Treatment Planning, Plan Evaluation, and Approval

**Figure 5:**
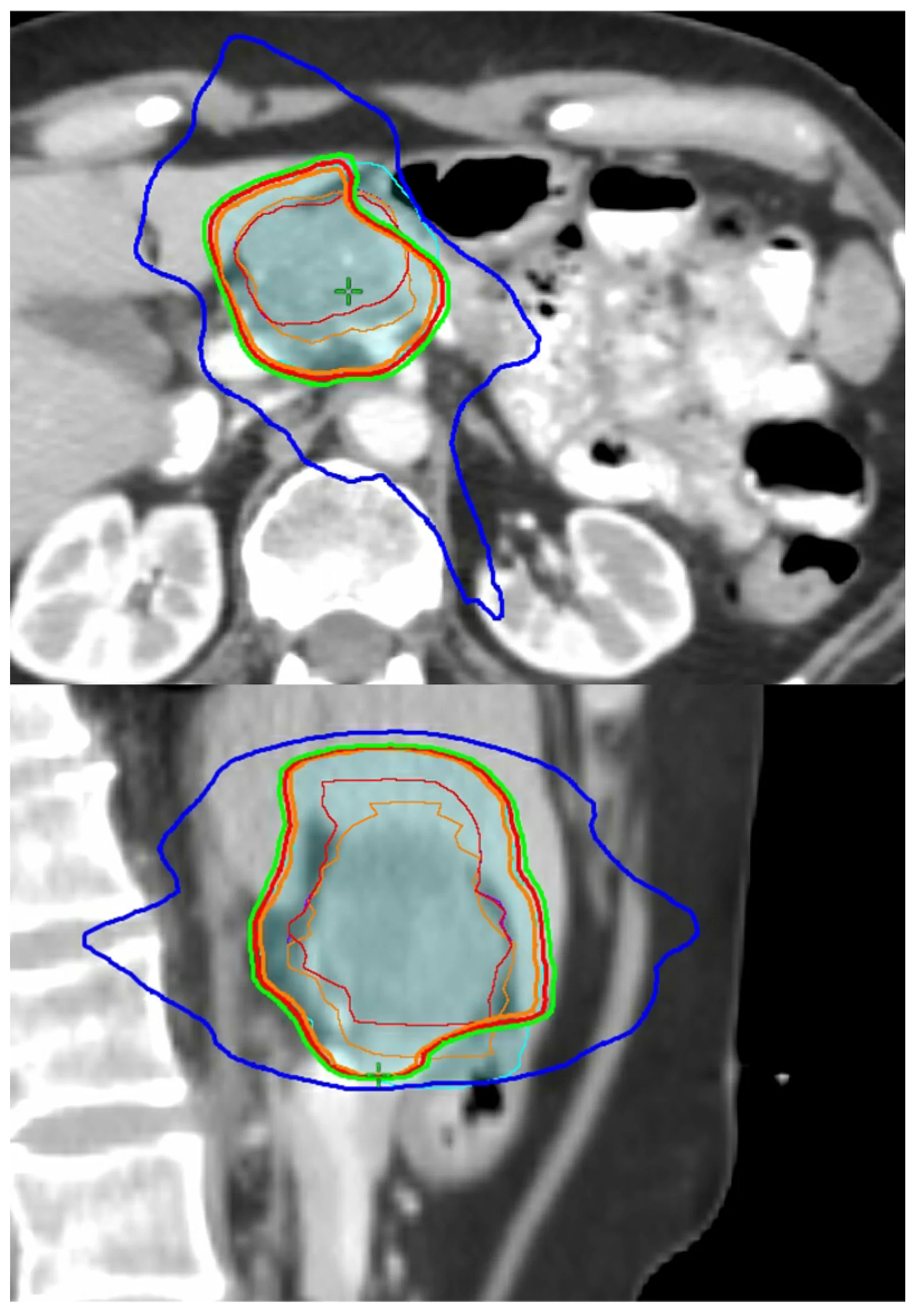
SBRT plan for the treatment of pancreatic cancer. The targets are GTV-Expiration (red), GTV-Inspiration (orange), ITV (shaded area in violet) and PTV (cyan). Isodose distributions are orange: 3300 cGy (100%), Red: 3135 cGy (95%), Green: 2970 cGy (90%), Blue: 1650 cGy (50%). Duodenum (pink).

Treatment plans are evaluated for target coverage and normal tissue dosing. The plan should aim to cover 100% of the GTV and >90% of the PTV by the prescription isodose whenever possible. D_max_ should be within the PTV and <130–150% of the prescription dose. The planning constraints should be met whenever possible. Any violations of dose-volume constraints should be discussed with the SBRT team and approved. The D_max_ at central hot spots should be <130–150% (Figure 5).

## 12. Plan Normalization

The treatment plan should be initially normalized such that 100% of the dose corresponds to the maximum dose within the PTV. While this point will typically correspond to the PTV centre of mass, it can be located elsewhere within the PTV. The prescription isodose surface will be chosen such that 95% of the target volume (PTV) is conformally covered by the prescription isodose surface. Doses less than 95% of the prescription dose are restricted to the outside edges of the PTV. A multileaf collimator for conformal or intensity modulated treatments is required. Flattening filter free, volumetric modulated arc therapy, inverse planned IMRT, forward planned IMRT, and conventional 3D CRT are permitted.

## 13. Mock Set up and Treatment with Image-Guidance

**Figure 6:**
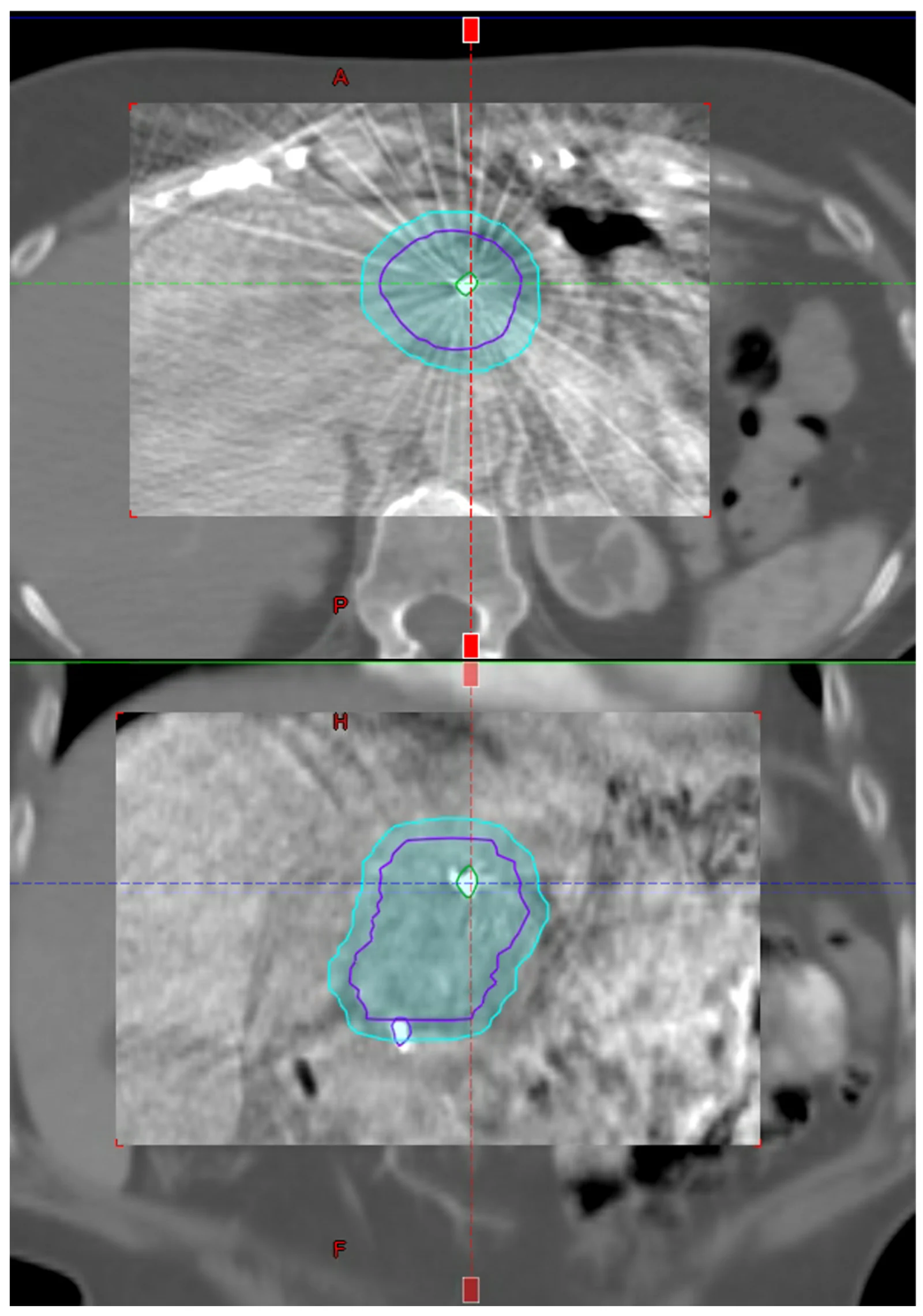
Cone beam CT of a patient with match of fiducials. The targets are ITV (shaded area in violet) and PTV (cyan). The green contour is a fiducial seed.

**Figure 7:**
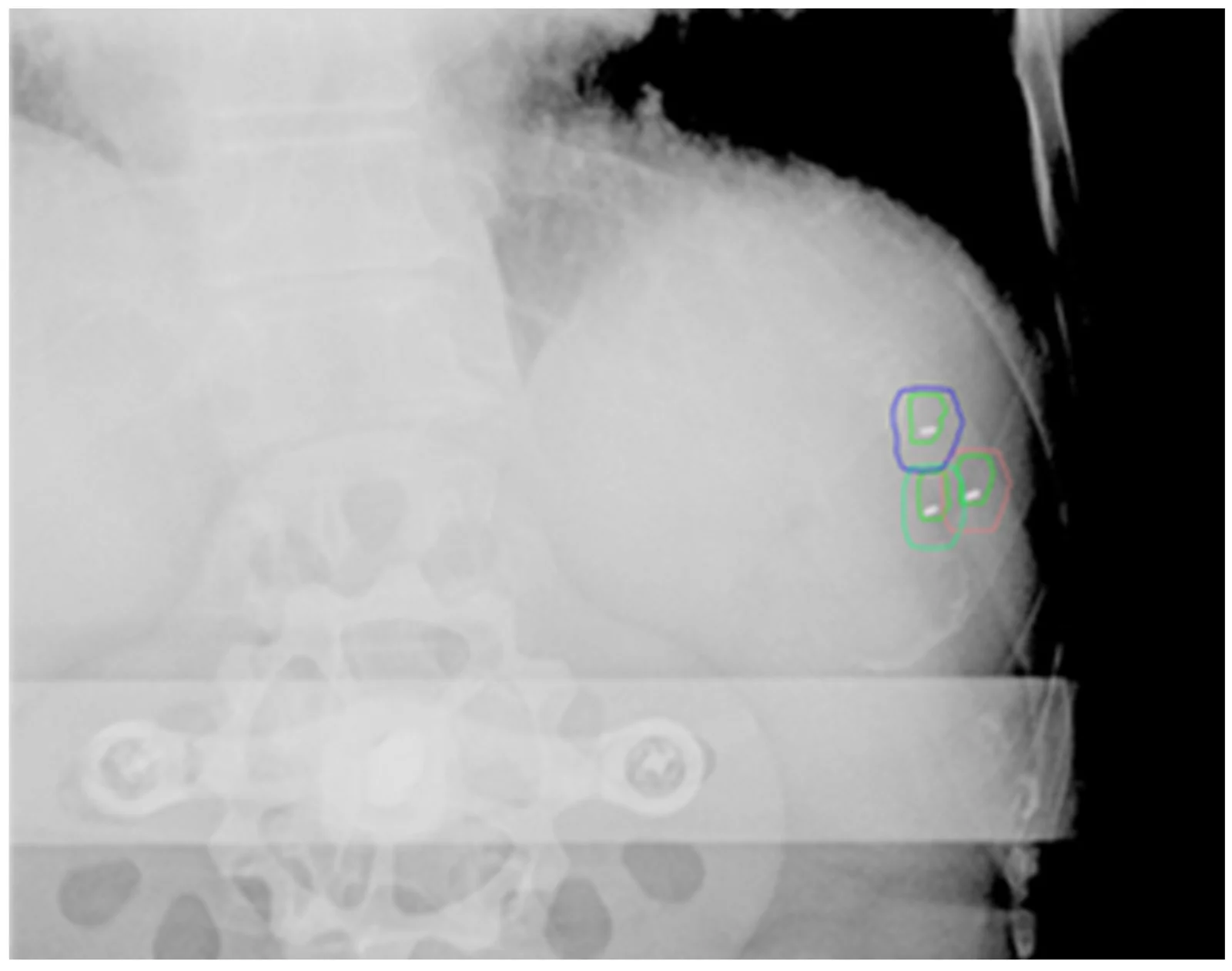
A 2D image confirming that the fiducials are located within the predefined motion contours (blue, pink, green), the smaller green contours are fiducial contours defined on the MIPs.

Each patient will have a mock set up appointment prior to treatment. The SBRT team, including the radiation oncologist, medical physicist, and dosimetry team, should be present at the mock set up. The purpose of this session is to assess immobilization and evaluate motion management and the patient’s ability to follow instructions for the smooth delivery of the proposed treatment. Cone beam CT (CBCT) is initially matched with the fiducials (Figure 6), followed by inspection of the pancreas and the surrounding soft tissue match prior to therapy. The CBCT will be acquired with gated breath-hold if the patient is being treated at exhale breath-hold. Fluoroscopy at anterior-posterior (AP) and lateral views to visualize the position of the fiducials through the breathing cycle (if under compression) or at exhale (if breath-hold) is useful to confirm they are within the predefined motion contour (Figure 7). If the fiducials are seen to move outside the motion contour, an estimate of the excursion outside of the contours is made during the fluoroscopic image acquisition and couch shifts are applied accordingly using the 2D–2D match software. A repeat fluoroscopy is then acquired to ensure the applied shifts are appropriate. If the shifts based on the fluoroscopy images are greater than tolerance (e.g., >2 mm), a repeat CBCT is required to confirm the soft tissue match is still acceptable.

Real-time motion management strategies during treatment consist of using an external and/or internal surrogate. The external surrogate is a respiratory block that can be placed on the patient’s abdomen and is monitored continuously by in-room IR cameras. This provides real-time phase and amplitude information. For gated and EEBH treatments, the linear accelerator can be programmed such that the beam is only allowed to turn on within predefined phases or amplitudes. An internal surrogate provides additional monitoring capabilities via kV imaging of fiducials. For FB and compression treatments, kV images can be taken every few seconds (typically every 3 s), during beam delivery. For gated treatments, kV images can be triggered at the beginning of each gating window. In the case of EEBH, kV images can be triggered at the beginning of each breath hold. Modern linacs have motion management functions (e.g., Auto-Beam Hold feature on the Varian Truebeams) that can be enabled such that the beam will turn off if the fiducials are detected outside of their predefined motion contour. Post-treatment fluoroscopic kV imaging is acquired at one angle as a record of patient position once treatment is complete.

## 14. Toxicity

SBRT to the pancreas is generally well tolerated and the strict adherence to normal tissue dose constraints render serious toxicity unlikely (31). Acute toxicities may be more pronounced 1–2 weeks after SBRT and these could lead to deterioration of performance status. The common side effects related to SBRT are fatigue, nausea, and vomiting. Other reported GI system-related acute toxicities include irritation, ulceration, or bleeding of the stomach, duodenum, small bowel, or large bowel. Fistulas, obstruction, or changes in motility following therapy can also occur but they rarely cause bowel perforation. To minimise toxicity, patients should be provided antiemetics (e.g., ondansetron) 30 min prior to radiation and a proton pump inhibitor is recommended for a period of 2–4 months.

## 15. Limitations

The primary limitation of our review is that there is no discussion on the workflow of other SBRT platforms such as robotic SBRT by CyberKnife or Stereotactic Magnetic Resonance-Guided Adaptive Radiation Therapy (SMART) using Linac-MRIs, which are increasingly used worldwide. The robotic Linac on the CyberKnife can actively track the patient’s fiducial motion via continuous imaging by its dual X-ray system to further reduce ITV margins. SMART technology represents one of the most recent significant innovations in pancreatic radiotherapy which allows direct visualization of the tumour (i.e., no fiducials) and OARs during RT and allows daily online adaptive dose-escalated radiotherapy [58]. Linac-MRIs can apply similar motion management strategies such as EEBH, compression, and gating discussed in our workflow, using cine MRI instead of kV. Massaccesi et al. reported the feasibility of OAR avoidance with MRI-guided respiratory-gated IMRT [59], and Rudra et al. investigated the use of a SMART platform to treat PDAC [60]. The study demonstrated dose escalation without significant toxicity. The benefit of SMART-based dose-escalated RT following induction chemotherapy needs prospective evaluation with respect to local control and OS without increasing toxicity. Despite their advantages, SMART/CyberKnife treatments are significantly more resource intensive and less accessible compared to our presented workflow using standard Linacs using EEBH, compression, or gating techniques to manage motion.

## 16. Conclusions

The treatment of pancreatic cancer incorporating SBRT has evolved over the last few years. Pancreatic SBRT appears to have a significant role in achieving local control and for improving survival outcomes. Precise treatment planning and delivery with SBRT has reduced the incidence of acute or late toxicity without compromising loco-regional control [43]. Future phase 3 studies integrating SBRT along with neoadjuvant chemotherapy are warranted to define the definite role of pancreatic SBRT.

## Figures and Tables

**Table 1:** Studies include radiotherapy for the treatment of pancreatic cancer.

Study, Year	Study Design	No of Patients	CRT/Neo-Adjuvant CRT	RT Dose and Fractionation	Median Follow-Up in Months	Median OS (95% CI) in Months	Comments
Herman et al., 2008 [10]	Prospective database review	271/616	5-FU based CRT	3DCRT RT dose of 50.4 Gy and daily fraction of 1.8 Gy	17.8	Patients who received CRT had improved median OS compared to no CRT (21.2 vs. 14.4; *p* < 0.001)	CRT associated with improved survival (relative risk 0.74; 95% CI, 0.62 to 0.89).
Hsu et al., 2010 [11]	Prospective database review	496	5-FU based CRT	RT dose of 50.4 Gy and daily fraction of 1.8 Gy	18.8	Patients who received CRT had improved median OS compared to no CRT (21.1 vs. 15.5; *p* < 0.001)	Survival benefit for adjuvant chemoradiation with a relative risk of 0.59 (0.48–0.72)
LAP07, 2016 [12]	Ph. 3	442	Patients after 4 × GEM-based treatment randomised to CRT with cape to GEM-based CT	RT dose of 54 Gy and daily fraction of 1.8 Gy	42.5	21 vs. 14 (HR:0.064)	There were no differences in OS (median survival 15.2 months vs. 16.5 months) LRR was less frequent in the CRT group (32% vs. 46%).
RTOG 97–04, 2011 [13]	Ph 3, subset analysis	451	CRT (50.4 Gy in 28 fractions) with pre and post 5-FU (n = 230) or GEM (n = 221).	50.4 Gy in 28 fractions	18	Median survival and 5-year OS of 20.5 months and 22% in GEM group vs. 17.1 months and 18% in 5-FU group (HR: 0.84; 95% CI: 0.67–1.05; *p* = 0.12)	Significantly fewer LRR in the CRT trial (35%) compared with the previously mentioned studies, despite a lower R0 resection rate in the RTOG 9704 trial (42%)
Jang et al., 2018 [14]	Ph. 2/3	50	27 patients were allocated to GEM based neoadjuvant CRT and 23 to upfront surgery groups	RT consisted of 45 Gy in 25 fractions and 9 Gy in 5 fractions		Median OS was significantly better in the neoadjuvant chemoradiation group than the upfront surgery group (40.7%, 21 months vs. 26.1%, 12 months)	Patients who had neo-adjuvant CRT in BRPC achieved significantly higher R0 resection and better survival rates than those who had no adjuvant therapy [HR 1.495, *p* = 0.028] The study reported improved local control (32% vs. 46%, *p* = 0.03) and quality of life without an impact on overall survival
Versteine et al. 2022 [15]	Ph 3	246	119 patients neoadjuvant CRT followed by surgery and 127 patients to upfront surgery	RT consists of 36 Gy in 15 fractions	59 months	5-yr OS 20.5% (95% CI, 14.2 to 29.8) with neoadjuvant CRT and 6.5% (95% CI, 3.1 to 13.7) with upfront surgery	Neoadjuvant GEM-based CRT followed by surgery and adjuvant GEM improves OS compared with upfront surgery and adjuvant GEM in resectable and BRPC

Abbreviations: CRT: chemoradiotherapy; CI: confidence interval; 5-FU: 5-flurouracil; 3DCRT: 3-dimensional conformal radiotherapy; GEM: gemcitabine; n: number of patients.

**Table 2:** ITV generation based on motion management techniques.

Motion Management Technique	ITV Generation Method
Free Breathing	Contour GTV Expiration and End Inspiration and merge
Compression	
Breath Hold	No motion. ITV = GTV
Gating	(1) Contour End Expiration GTV, expand by 5 mm in all directions except superior (2) Contour GTV on all phases of gating window and combine (3) Contour End Expiration GTV, apply custom expansion based on measurement of fiducial motion in all 3 planes during gating window.

**Table 3:** Organs at risk (OARs) constraints for planning pancreatic SBRT.

Dose/No of Fractionations (Ref)	OARs	Dose Constraints
33 Gy/5 Herman et al. [28]	Luminal OARs: Liver Spinal cord Kidneys (combined)	9 cc: < 15 Gy; 3 cc: < 20 Gy; 1 cc: < 33 Gy 50%: < 12 Gy 1 cc: > 8 Gy 75%: < 12 Gy
25–40 Gy/5 Alliance trial A021501 [56]	Luminal OARs: Liver: Combined kidneys: Spinal cord:	V15 < 9 cm^3^; V20 < 3 cm^3^; V33 < 1 cm^3^ V12 < 50% V12 < 75% V20 < 1 cm^3^
33–40 Gy/5 Rhee [57]	Luminal OARs: Liver: Spinal cord: Combined Kidneys:	D_max_ < 40 Gy; D1.0 cc < 35 Gy; D10.0 cc < 30 Gy; D30.0 cc < 20 Gy D_max_ < 55 Gy; D50% < 12 Gy D_max_ < 20 Gy D25% < 12 Gy
33 Gy/5 (ASTRO) [53]	Luminal OARs: Liver: Spinal cord: Combined Kidneys:	V15 Gy < 9 cc; V20 Gy < 3 cc; V33 Gy < 1 cc V12 Gy < 50% V20 Gy < 1 cc V12 Gy < 75%
40 Gy/5 AGITG/TROG [52]	Luminal OARs: Liver: Spinal cord PRV: Combined Kidneys:	V33 < 0.5 cc; V30 < 5 cc V12 < 40% V20 < 0.5 cc V12 < 25%
50 Gy/5 Grimbergen et al. [55]	Luminal OARs: Small bowel: Liver: Spinal cord: Combined Kidneys:	0.5 cc <V35 Gy 0.5 cc < 40 Gy 0.5 cc < V40 Gy 0.1 cc < V28 Gy D_mean_ < 10 Gy; D67% < 16.8 Gy

Abbreviations: Luminal OARs: Duodenum/stomach/small Bowel/large bowel.

## Data Availability

No new data were created or analyzed in this study. Data sharing is not applicable to this article.

## Supplementary Materials

The following supporting information can be downloaded at: https://www.mdpi.com/article/10.3390/curroncol33090519/s1, Table S1: Studies using SBRT following induction chemotherapy or along with concurrent chemotherapy in the treatment of pancreatic cancer.
