# Supplementary material for: Practical Considerations in the Radiotherapy Treatment Planning for SBRT Pancreas Program
Source: Curr Oncol. 2026 Aug 31;33(9):519. doi: 10.3390/curroncol33090519 (PMC13605244; doi:10.3390/curroncol33090519)
Supplement: Supplementary file 1 [file curroncol-33-00519-s001.zip › curroncol-4420835-supplementary.pdf]

Supplementary Table S1. Studies using SBRT following induction chemotherapy or along with concurrent chemotherapy in the treatment of pancreatic cancer.

Study, year	Study design	No of patients	Induction(I)/ Concurrent(C) chemotherapy	SBRT dose in Gy / fractionation	SBRT Technique	Median OS (95% CI) in months	Median PFS in months	12 months Local control %	Resection %	Toxicity
Tozzi et al. 2013 [29]	Ph 1	31	GEM/ GEMOX /GEM-5FU	45 Gy /6	VMAT	11	8	96	0	No G ≥3 RTX-related
Simoni et al. 2021 [30]	Ph1	59	FOLFIRINOX /GEM/nab- paclitaxel	50 Gy/5	VMAT/ Tomotherapy	10	11	Resected: 85 Non-resected:80	BRPC: 89 %, LAPC: 34 % (57.7 % R0)	No G ≥3 RTX-related
Hill et.al. 2022 [31]	Ph 2	48	FOLFIRINOX /GE/ GTX /nab-paclitaxel (GnP)	33Gy/5	NA	20.2	13	23.9	39 %, (75 % R0)	2.1 % late grade 2 +
van 't Land et al. 2023 LAPC-2 trial [32]	phase 1/2	38	FOLFIRINOX vaccinations with IMM-101	40 Gy/5	Cyberknife	19	12		21 % (75 % R0)	34 % G 3, no G 4 3 % G 5 none related to IMM-101.
Bordeau et al. 2022 [33]	Observational	70	FOLFIRINOX / GEM-ABRAXANE/FOLFOX /GEMCITABINE /FOLFIRI	50/5	MR guided	21		87	39	2 % acute G3, 4 % late G3
Jung et al.,2019 [34]	Retrospective review	95	FOLFIRINOX/GE based	28/4	IMRT/VMAT	16.7	10.2	NA		3.2%acuteG3+ 3.2% late G3+
Herman et al., 2015 [28]	Ph 2	49	GE	33 Gy/5	variable	13.9	7.8	78	8	2%acuteG2+ 12%lateG2+
Shen et al., 2019 [35]	retrospective	56	GE+ capecitabine	40/5	Cyberknife	19	12	NA		3.6%acuteG3 5.4%lateG3 3.6%lateG4
Kim et al., 2019 [36]	Observational	27	FOLFIRINOX	25-42/3-5	VMAT	11.6		67		
Comito et al ,2017 [37]	Ph 2	45	GEMOX / GEM / PEF- G /Altro	45/6	IMRT	13	8	87		Acute: 29% Grade ≥ 2 Late: 3% Grade ≥ 2
Liauw et al. 2020 [38]	Ph 1/2	15	FOLFIRINOX /GEM	30-45/3	variable	12.8	7	80		Acute: 53% Grade ≥ 2 Late: 27% Grade ≥ 2
Teriaca et al. 2021 [39]	Ph 2	39	FOLFIRINOX	40/5	variable	18	11	81	18	Acute: 10% Grade ≥ 3 Late: 3% Grade ≥ 3

Parikh et al. 2023 [40]	Ph 2	136	FOLFIRINOX / GE/nab-paclitaxel	50/5	SMART/MR guided	Not reached		83	All: 32 % BRPC: 56 % LAPC: 14 %	7 % ≥3 G3 GI toxicity
Reyngold et al,2023 [41]	Ph 1	24	FOLFIRINOX /GE-nab-paclitaxel	33-37/3	IMRT	11		75		Acute: 0% Grade ≥3 Late: 0% Grade ≥3

Abbreviations: BRPC: borderline resectable pancreatic cancer; CTx: Chemotherapy; Dx: Diagnosis; FOLFIRINOX: oxaliplatin +folinic acid +irinotecan +fluoro uracil; FOLFIRI: Leucovorin +irinotecan +fluorouracil; FOLFOX: Leucovorin +irinotecan +Fluorouracil, G =Grade; FFLP: Freedom from local progression; GI: Gastrointestinal; Gy: Gray; LAPC: Locally advanced pancreatic cancer; LC: Local control; LPFS: Local progression-free survival; mPC: metastatic pancreatic cancer; RPC: resectable pancreatic cancer; NR: not reported; OS: Overall survival; PDAC: Pancreatic ductal adenocarcinoma; PFS: Progression-free survival; R0: negative resection margin; RT: Radiotherapy; SBRT: Stereotactic body radiotherapy; IMRT, Intensity-Modulated Radiation Therapy; NR, Not Reported; LAPC, Locally Advanced Pancreatic Cancer; BRPC, Borderline Resectable Pancreatic Cancer; SMART, Stereotactic Magnetic Resonance-Guided Radiation Therapy ;Gy=Grays; OS=overall survival; PFS=progression free survival; G=grade;N/A=notapplicable;GTX=gemcitabine+docetaxel+capecitabine;FOLFIRINOX=oxaliplatin+folinicacid+irinotecan+fluo rouracil;GE=gemcitabinealoneorgemcitabine+nab-paclitaxel;FOLFOX=oxaliplatin,folinicacid,andfluorouracil
